# Lung ultrasound can be used to predict the potential of prone positioning and assess prognosis in patients with acute respiratory distress syndrome

**DOI:** 10.1186/s13054-016-1558-0

**Published:** 2016-11-30

**Authors:** Xiao-ting Wang, Xin Ding, Hong-min Zhang, Huan Chen, Long-xiang Su, Da-wei Liu, Chinese Critical Ultrasound Study Group (CCUSG)

**Affiliations:** Department of Critical Care Medicine, Peking Union Medical College Hospital, Peking Union Medical College, Chinese Academy of Medical Sciences, Beijing, 100730 China

**Keywords:** Acute respiratory distress syndrome, Prone position, Lung ultrasound

## Abstract

**Background:**

It is very important to assess the effectiveness of prone positioning (PP) in patients with severe acute respiratory distress syndrome (ARDS). However, it is difficult to identify patients who may benefit from PP. The purpose of this study was to investigate whether prone positioning potential (PPP) can be predicted by lung ultrasound in patients with ARDS.

**Methods:**

In this prospective study, 45 patients with ARDS were included for the assessment of PPP. A PP lung ultrasound examination (PLUE) protocol was performed in the dorsal regions of the lung in 16 areas at H0, H3, and H6 (0, 3, and 6 h after PP). The ultrasonography videos were blindly evaluated by two expert clinicians to classify the lung regions as normal pattern (N), moderate loss of lung aeration (B1), severe loss of lung aeration (B2), and consolidation (C). The aeration scores were collected at H0, H3, and H6. According to the ratio of partial pressure of arterial oxygen to fraction of inspired oxygen (P/F ratio) at 7 days, patients were classified into PPP-positive (P/F ratio >300) and PPP-negative groups; also, the patients were classified into survival and nonsurvival groups according to 28-day mortality.

**Results:**

Aeration scores was compared at H0, H3, and H6. The scores were significantly reduced between H3 and H0, but there was no difference between H3 and H6. The aeration score variation (ASV) of the PPP-positive group between H3 and H0 was significantly higher than that in the PPP-negative group, and the sensitivity and specificity of ASV ≥5.5 for the PPP-positive group were 73.9% and 86.4%, respectively. The area under the receiver operating characteristic curve (AUROC) was 0.852 for the ASV. The ASV between H3 and H0 in the survival group was significantly higher than in the nonsurvival group. The sensitivity and specificity of ASV ≥7 for survival were 51.5% and 75%, respectively. The AUROC was 0.702 for the ASV.

**Conclusions:**

The PLUE protocol can be used to predict PPP and assess prognosis in patients with ARDS.

## Background

Prone positioning (PP) has been widely accepted as one of the important therapeutic strategies for acute respiratory distress syndrome (ARDS) [1]. Although the results of several randomized controlled trials have shown that PP could improve oxygenation and reduce 28-day mortality in patients with ARDS [2], it is also accompanied by several risks, such as the increasing risk of unintended extubation and hemodynamic disturbance [3]. So, it seems to be very important to assess the effectiveness of PP in patients with severe ARDS and to predict whether these patients can benefit from this procedure.

Unfortunately, there is no effective method to monitor aeration improvement at the bedside during PP. Computed tomography (CT) is an effective way to observe the aeration of the dependent regions during PP [4], but it cannot be used in daily practice. Recently, the quick development of lung ultrasound (LUS) has provided a new way to evaluate lung aeration at the bedside for critically ill patients. As when patients are in PP, scanning the anterior regions of the lung requires a second person to lift the shoulder of the patient, which is not convenient in daily practice [5]. In this study, LUS was applied in the dorsal regions of patients receiving PP ventilation, and its values in detecting the changes of lung aeration during PP were evaluated. In addition, a new concept, “prone position potential” (PPP), was developed to predict whether patients could benefit from PP.

## Methods

### Participants

All of the patients with ARDS admitted to the Department of Critical Care Medicine, Peking Union Medical College Hospital (a 30-bed intensive care unit [ICU] in a tertiary hospital), from October 2012 to February 2014 were screened for inclusion this prospective study. The inclusion criteria were moderate to severe ARDS (fulfill the 2011 Berlin definition of ARDS [6]), intubated, and receiving mechanical ventilation for longer than 36 h. The exclusion criteria were massive subcutaneous emphysema or dressings in the examining area, as well as patients with do not resuscitate orders. This study was approved by the ethics committee of the Peking Union Medical College Hospital (S-617), and written informed consent was provided by the next of kin of all subjects.

Lung-protective ventilation was applied to all patients according to a standardized protocol. Mechanical ventilation was delivered in a volume-controlled mode with constant inspiratory flow, with tidal volume (V_t_) targeted at 6 ml/kg of predicted body weight [7] and the positive end-expiratory pressure (PEEP) level selected from a PEEP-fraction of inspired oxygen (FiO_2_) table [2]. Patients were placed in PP twice daily, at least 6 h per time. Sufficient sedation and analgesia were provided, and neuromuscular blocking agents were used as needed. Continuous heart rate, blood pressure, and transcutaneous oxygen saturation were monitored.

### Lung ultrasonographic assessment

**Fig. 1:**
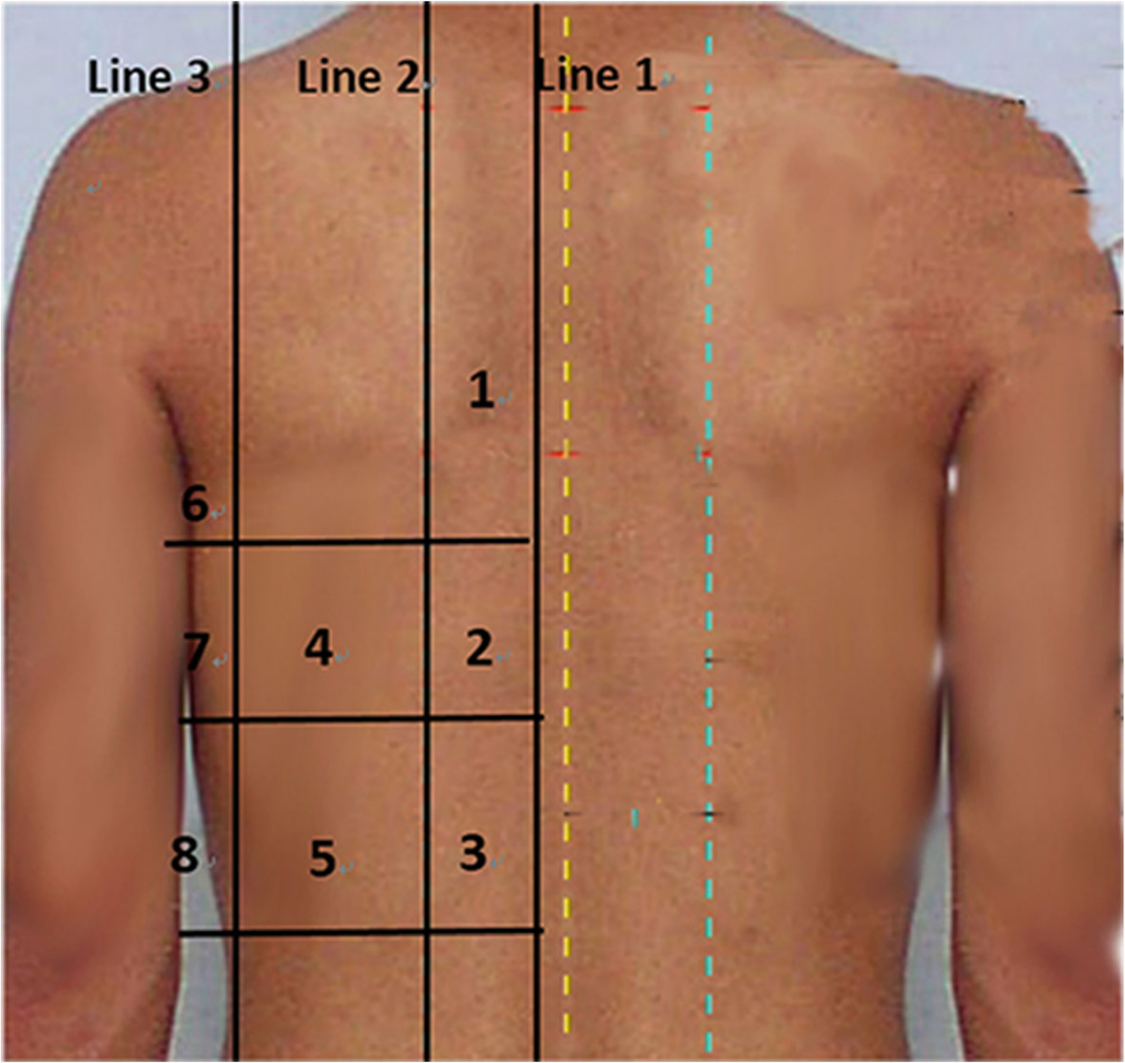
The examination areas in the prone position lung ultrasound examination protocol. *Line 1* paravertebral line, *line 2* scapular line, *line 3* posterior axillary line

An M-Turbo ultrasound machine and a 2- to 5-MHz curved array probe (FUJIFILM SonoSite, Bothell, WA, USA) were used for all the examinations. A prone position lung ultrasound examination (PLUE) protocol was applied at H0 (immediately after PP was started) as well as at H3 and H6 (3 and 6 h, respectively, after PP was started) during the first PP. The examination points were as follows: the paravertebral line, scapular line, and posterior axillary line were used as body markers to divide the back of the single side into 3 regions, then every region was divided into 3 equal areas to get 9 examination areas, 8 points (the point covered by the scapular bone was ruled out) in the single side and 16 points in total for both sides (Fig. 1).

The sonographic signs of lung aeration were classified into four categories: (1) normal pattern (N): presence of lung sliding with A lines or isolated B lines (less than three); (2) moderate loss of lung aeration (B1): multiple spaced B lines; (3) severe loss of lung aeration (B2): coalescent B lines; and (4) consolidation (C): the presence of a tissue pattern characterized by dynamic air bronchograms. For a given region of interest, points were allocated according to the worst ultrasound pattern observed: N = 0, B1 lines = 1, B2 lines = 2, and C = 3. The lung scores were calculated as the sum of points [8]. The aeration score variation (ASV) was defined as the difference between the scores at different time points (H3 vs. H0, H6 vs. H3).

Each LUS video recording was retrospectively evaluated and scored anonymously and blindly by two ICU expert physicians who were certified for the critical LUS. The intra- and interobserver reliability were optimum for video recordings, with kappa values of 0.82 and 0.77, respectively.

### Ratio of partial pressure of arterial oxygen to fraction of inspired oxygen and dead space measurements

Air-blood gas and end-tidal carbon dioxide (ETCO_2_) were analyzed separately at H0, H3, and H6. The ratio of partial pressure of arterial oxygen to fraction of inspired oxygen (P/F ratio) was used to evaluate the oxygenation changes in the procedure. The Bohr equation, V_d_/V_t_ = (PaCO_2_ − ETCO_2_)/PaCO_2_, where PaCO_2_ is partial pressure of arterial carbon dioxide, was used to calculate the percentage of dead space (V_d_).

### Prone positioning potential definition

The PPP was defined according to the P/F ratio 7 days after the therapy. The patients with a P/F ratio ≥300 mmHg were classified as PPP-positive, and those with a P/F ratio <300 mmHg were classified as PPP-negative.

### Statistical analysis

SPSS 16.0 software (SPSS, Chicago, IL, USA) was used for the analysis. Results for continuous variables with normal distributions, including age, Acute Physiology and Chronic Health Evaluation II score, and lung aeration score are given as means ± SD. Student’s *t* test was used to compare means between two groups. Results for continuous variables that were not normally distributed are given as medians (25th and 75th percentiles) and were compared using nonparametric tests. Paired and unpaired Student’s *t* tests were used to compare quantitative variables, such as ASV. The discriminatory power of the ASV scores was quantified by measuring the area under the receiver operating characteristic curve (AUROC). Pearson’s correlation test was used for bivariate correlation analysis. The intra- and interobserver reliability for video recordings were calculated by kappa test of variance for consistency.

## Results

### Participants

**Fig. 2:**
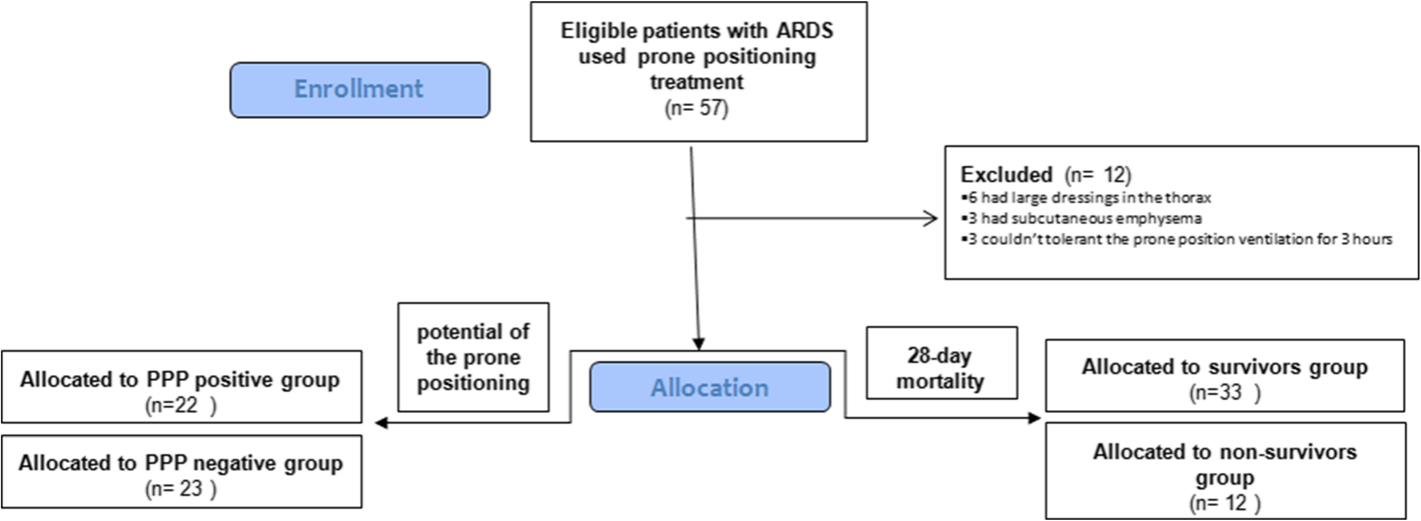
Flowchart of the study. *ARDS* Acute respiratory distress syndrome, *PPP* Prone positioning potential

Fifty-seven consecutive patients were enrolled in the study. Of these, 12 patients were excluded: 6 had large dressings in the thorax, 3 had subcutaneous emphysema, and 3 could not tolerant the PP ventilation for 3 h because of alterations in hemodynamics (1 experienced tachycardia and the other 2 had remarkable hypotension). Forty-five patients were ultimately included in the study (Fig. 2). The characteristics of the population are outlined in Table 1.

**Table 1:** Characteristics of patients with acute respiratory distress syndrome in this study

Characteristics (*n* = 45 patients)	Data
Sex ratio, male/female	32/13
Age, years	65.0 ± 17.2
APACHE II score	16.9 ± 8.2
Tidal volume, ml/kg of predicted body weight	5.6 ± 0.6
Initial PEEP, mmHg	9 ± 2
Initial P/F, mmHg	132 ± 32
Prone positioning duration, h	42 ± 16

*Abbreviations: APACHE* Acute Physiology and Chronic Health Evaluation, *PEEP* Positive end-expiratory pressure, *P/F* Ratio of partial pressure of arterial oxygen to fraction of inspired oxygen

Thirty-five (77.8%) of 45 of these patients with ARDS had severe pneumonia, and 10 (22.2%) of 45 had ARDS secondary to septic shock. Of all 45 patients, 12 (26.7%) of the patients had died by day 28. Additionally, according to the PPP definition, 51.1% (23 of 45) of the patients had a P/F ratio ≥300 mmHg 7 days after the first PP therapy and were classified into the PPP-positive group, and 48.9% (22 of 45) of the patients had a P/F ratio <300 mmHg and were classified into the PPP-negative group.

### Lung aeration scores

**Fig. 3:**
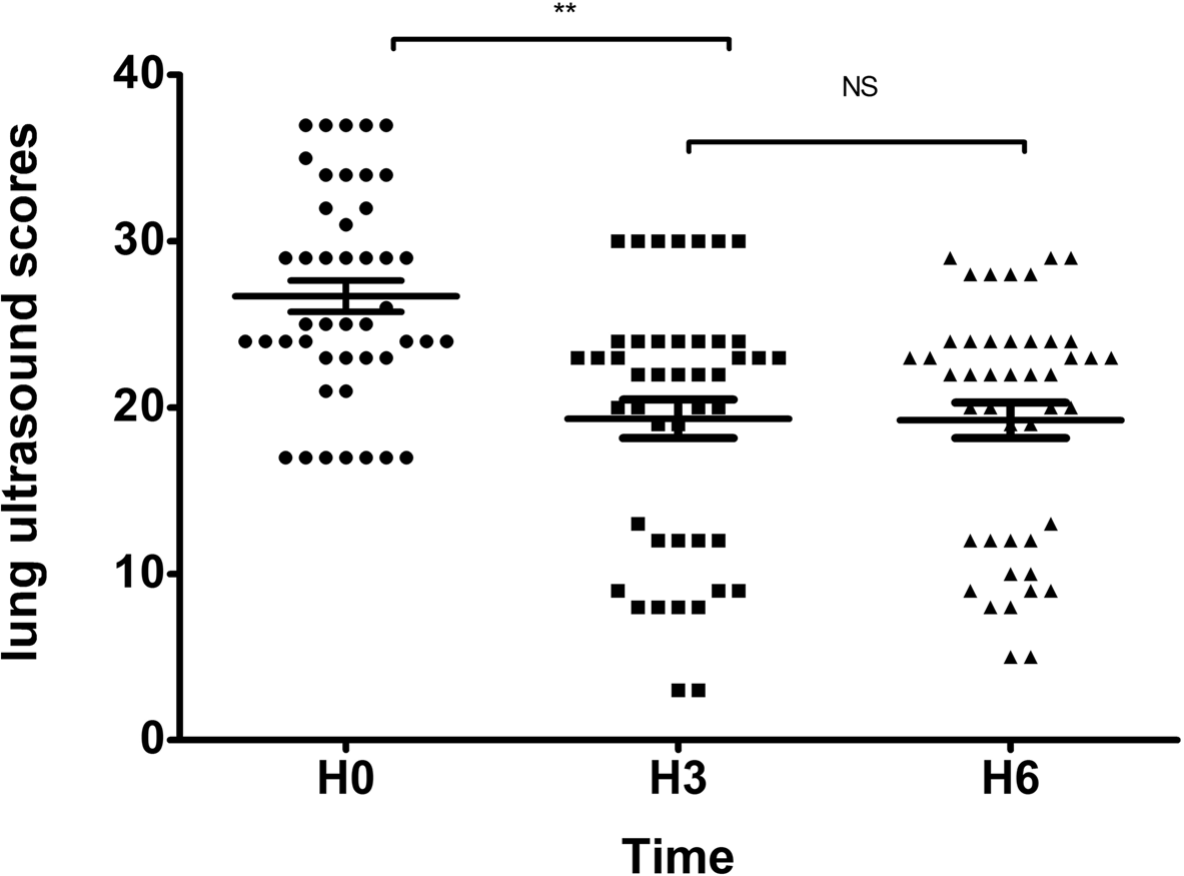
Lung ultrasound scores of the dependent region at different time points (** *p* < 0.01). *NS* No significant difference. *H0* Start of prone positioning, *H3* 3 h after prone positioning, *H6* 6 h after prone positioning

At H0, the LUS scores of the PPP-positive group and the PPP-negative group were 23.8 ± 6.5 and 29.7 ± 5, respectively (*p* > 0.05), and the LUS scores of the survival group and nonsurvival group were 26.3 ± 6.5 and 27.7 ± 5.9, respectively (*p* > 0.05). There were no significant differences between the groups. At H3, the LUS score of the lung-dependent region was significantly reduced compared with H0 (19.3 ± 7.8 vs. 26.7 ± 6.3; *p* < 0.001), but at H6, there was no significant reduction of the LUS score compared with H3 (19.3 ± 7.8 vs. 19.2 ± 7.2; *p* = 0.511) (Fig. 3). Because there was no significant difference between the LUS scores at H3 and H6, the ASV between H3 and H0 was chosen for further analysis.

**Fig. 4:**
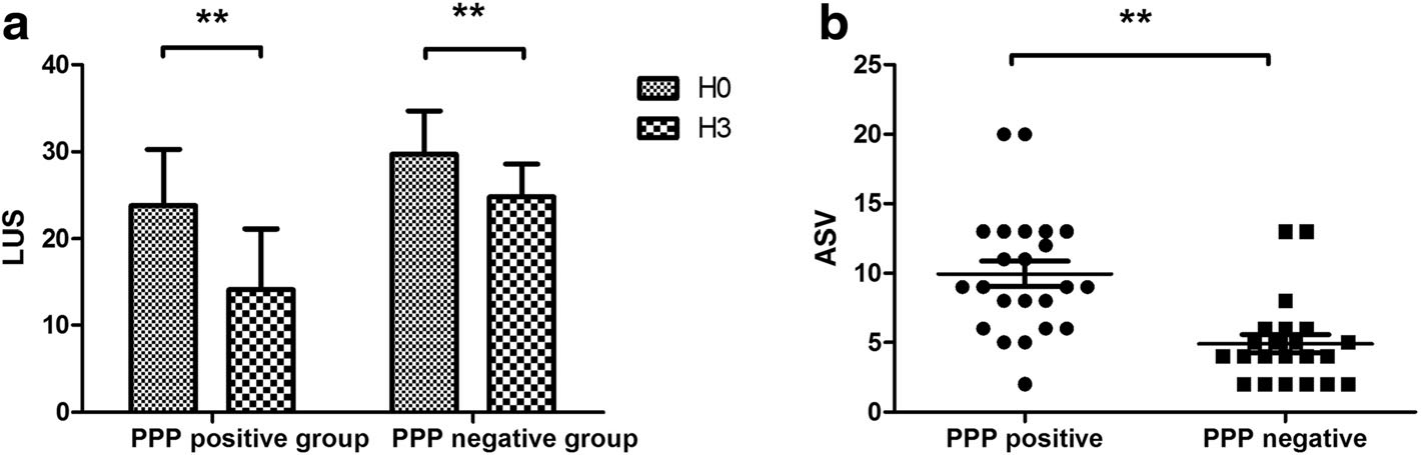
**a** Lung ultrasound scores at H0 and H3 in the PPP-positive and PPP-negative groups. **b** The aeration score variations between H3 and H0 in the PPP-positive and PPP-negative groups (** *p* < 0.01). *ASV* Aeration score variation, *H0* Start of prone positioning, *H3* 3 h after prone positioning, *LUS* Lung ultrasound score, *PPP* Prone positioning potential

**Fig. 5:**
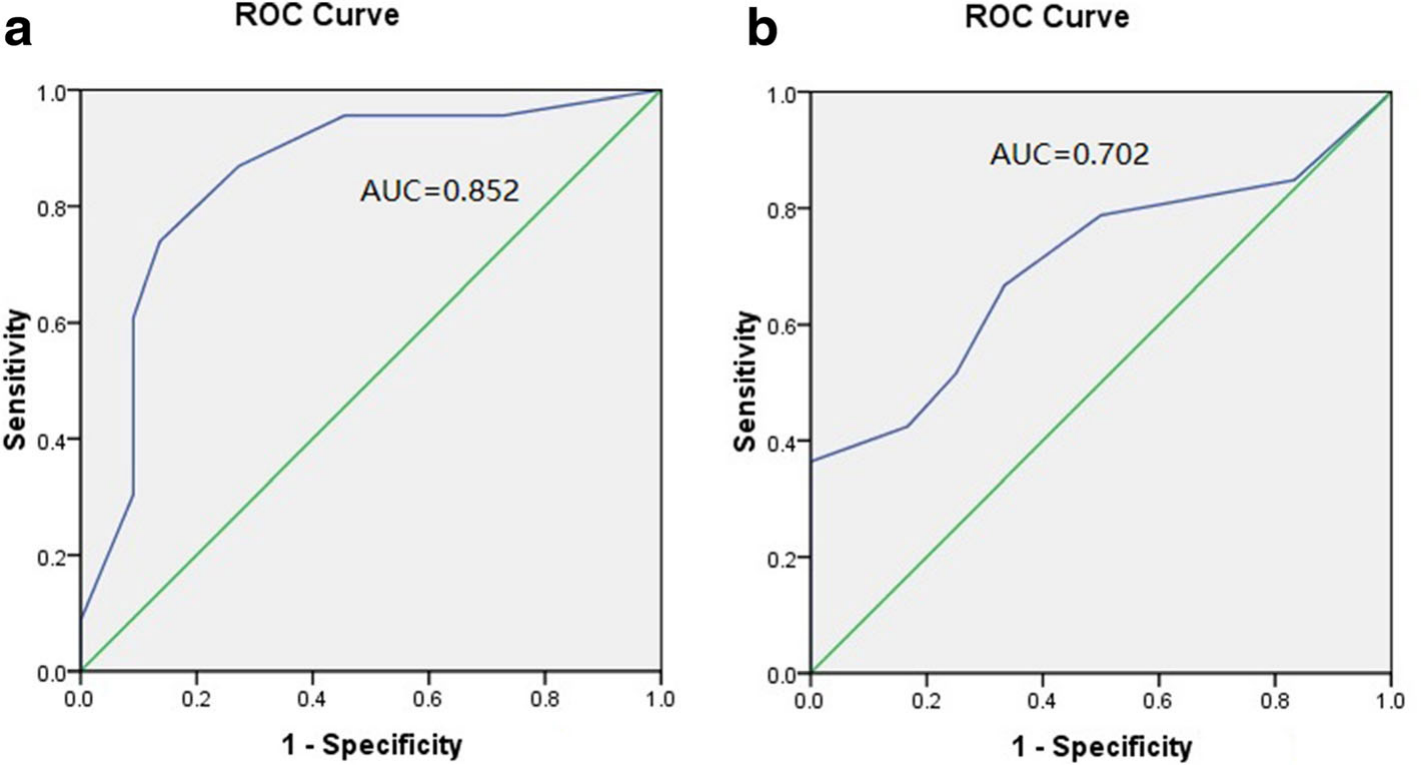
**a** Receiver operating characteristic curve (ROC) predicting the prone positioning potential with the aeration score variation (ASV). **b** ROC predicting the survival state with the ASV. *AUC* Area under curve

In the PPP-positive group, the LUS scores at H0 and H3 were 23.8 ± 6.5 and 14.1 ± 7.0, respectively (*p* < 0.01) (Fig. 4a, *left*); in the negative group, the LUS scores at H0 and H3 were 29.7 ± 5 and 24.8 ± 3.8, respectively (*p* < 0.01) (Fig. 4a, *right*). The ASV (H3 vs. H0) was 10.0 ± 4.4 in the PPP-positive group, significantly higher than that of the PPP-negative group (4.9 ± 3.1; *p* < 0.001) (Fig. 4b). The sensitivity and specificity of ASV ≥5.5 for the PPP-positive group were 73.9% and 86.4%, respectively. The AUROC for the ASV was 0.852 (95% CI 0.731–0.972) (Fig. 5a).

**Fig. 6:**
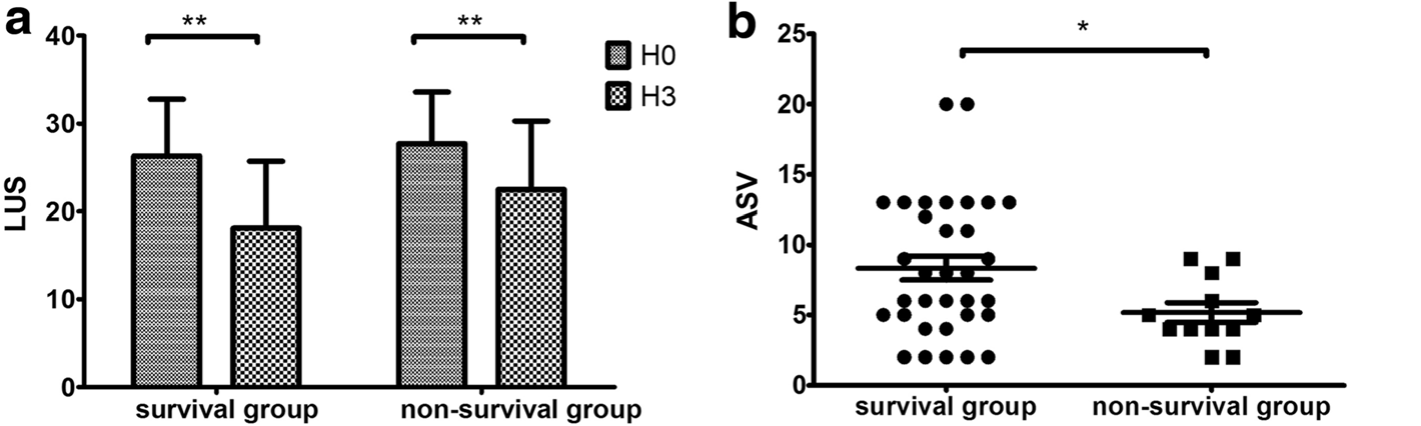
**a** Lung ultrasound scores at H0 and H3 in the survival and nonsurvival groups. **b** Aeration score variations between H3 and H0 in the survival and nonsurvival groups (** *p* < 0.01, * *p* < 0.05). *LUS* Lung ultrasound score, *ASV* Aeration score variation, *H3* 3 h after prone positioning, *H0* Start of prone positioning

In the survival group, the LUS scores at H0 and H3 were 26.3 ± 6.5 and 18.1 ± 7.6, respectively (*p* < 0.001) (Fig. 6a, *left*). In the nonsurvival group, the LUS scores at H0 and H3 were 27.7 ± 5.9 and 22.5 ± 7.8, respectively (*p* < 0.001) (Fig. 6a, *right*). The ASV between H3 and H0 in the survival group was significantly higher than in the nonsurvival group (8.3 ± 4.9 vs 5.2 ± 2.4; *p* < 0.05) (Fig. 6b). The sensitivity and specificity of ASV ≥7 for survival were 51.5% and 75%, respectively. The AUROC for the ASV was 0.702 (95% CI 0.547–0.857) (Fig. 5b).

### P/F ratio and dead space ventilation

**Fig. 7:**
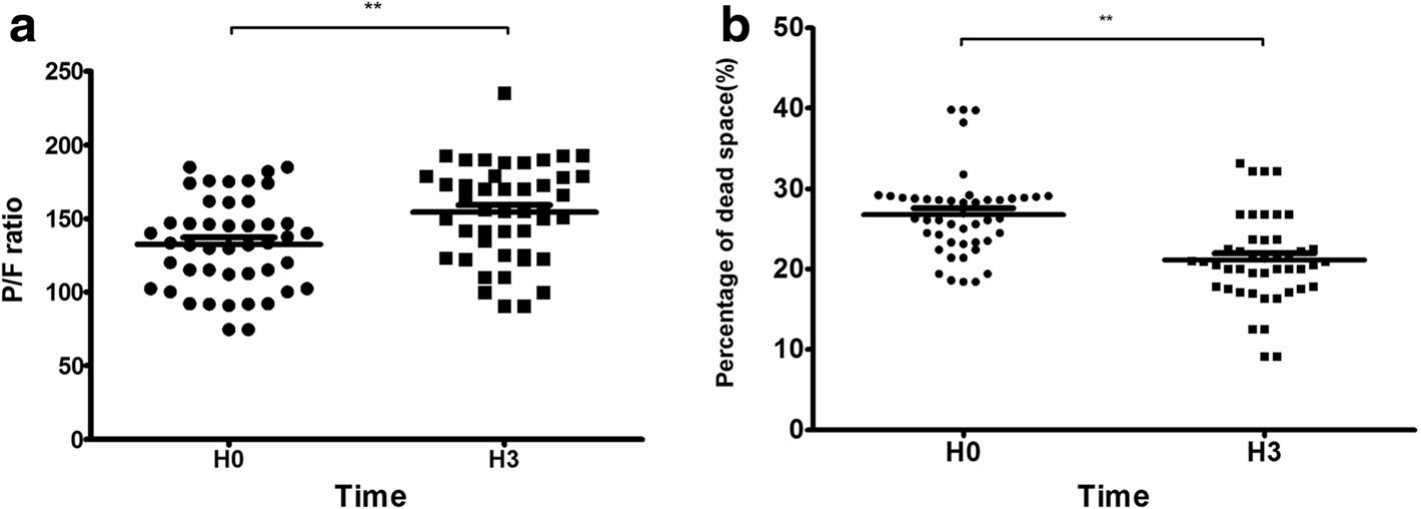
**a** The ratio of partial pressure of arterial oxygen to fraction of inspired oxygen (*P/F ratio*) during prone positioning at H0 and H3. **b** The percentage of dead space during prone positioning at H0 and H3 (** *p* < 0.01). *H3* 3 h after prone positioning, *H0* Start of prone positioning

**Fig. 8:**
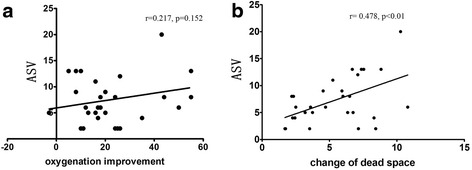
**a** Correlation of oxygenation improvement and aeration score variation (ASV). **b** Correlation of the change in dead space and ASV

Compared with H0, at H3 the P/F ratio was significantly improved (154.38 ± 32.47 vs. 132.47 ± 30.9; *p* < 0.01). In addition, the dead space was significantly reduced at H3 (21.1 ± 5.4% vs. 26.8 ± 5.4%; *p* < 0.01) (Fig. 7). There was no correlation between the oxygenation changes and the ASV at H0 and H3 (*r* = 0.217; *p* = 0.152), but the ASV and the reduction of the dead space had a tight correlation (*r* = 0.478; *p* < 0.01) (Fig. 8).

## Discussion

ARDS is a severe respiratory disease worldwide with a mortality of 40–60%. Recently, PP has gained increasing attention in the treatment of ARDS. Several studies have shown that PP could improve oxygenation [9, 10], reduce ventilation-induced lung injury [11], and decrease 28-day and 90-day mortality [2]. The beneficial effect of PP has not yet been elucidated. Proposed explanations are that PP increases functional residual capacity, redistributes perfusion along a gravitational gradient toward less-injured lung regions, alters regional diaphragm motion, and results in better secretion removal.

In the past several years, researchers in a few studies have investigated aeration changes during the PPP ventilation. Gattinoni et al. described that, when body position was changed from supine to prone, there was a dramatic redistribution of lung densities from the dorsal to the ventral visualized by CT [4]. Additionally, transesophageal echocardiography (TEE) has been used to observe the change in density area while patients were in PP [12]. However, both CT and TEE are not easily accessible in clinical practice. Transthoracic LUS is a noninvasive, reliable, and highly reproducible tool for assessing lung reaeration at the bedside. In our present study, a PLUE protocol based on the LUS was developed to detect the aeration of the dependent regions of the lung during PP in patients with ARDS, and it showed a significant improvement of lung aeration during PP, consistent with previous studies [5].

Because early trials in which the PP duration was short did not show any benefit with PP, the PP duration was at least 16 h in several later studies [2, 13, 14], but none of these studies explained mechanics or monitored lung reaeration. Wang et al. found that, in patients with severe ARDS, application of PP for 2–4 h could significantly improve pulmonary ventilation, but more than 4 h did not further improve lung aeration or P/F ratio [15]. We found a similar scenario in our study, which showed that the lung aeration did not significantly improve 6 h after PP compared with 3 h. One possible explanation is that the aeration improvement was only one of the benefits; other effects of PP, such as improvement of right ventricular function and better sputum draining, that may take longer also may have contributed to the improvement of the prognosis.

Turning images into numbers (semiquantitation) is the key to effective LUS assessment of changes in the overall state of lung aeration. LUS offered an appealing way to semiquantitatively describe regional aeration, rather than using only the global amount of lung air content. LUS score-quantified aeration changes observed in patients with ventilator-associated pneumonia upon initiation of antimicrobial therapy showed a tight correlation with CT measurements of lung aeration [8]. When compared with the pressure-volume curve method of assessing PEEP-induced lung recruitment in acute lung injury (ALI)/ARDS, the same score was accurate in detecting significant increases in lung aeration [16, 17]. During a successful spontaneous breathing trial, aeration changes measured by LUS scores may accurately predict postextubation distress [18]. In our present study, the ASV between H0 and H3 could detect aeration improvement during PP. Some patients who have a high potential for lung recruitment might actually have an immediate response to position changes that cannot be detect by the LUS in time, so the ASV may underestimate the real response in these patients because the reaeration could take place in 1 h after the PP [5].

Actually, up to now, there were few effective indices able to predict outcome results when PP was done at first. The possibility of directly quantifying lung aeration during PP offers a decisive advantage for predicting the outcome because compromised aeration is one of the critical pathophysiological factors. Because there are still risks of severe complications during the procedures, such as hemodynamic turbulence and unintended extubation, a new concept—PPP—based on the PLUE protocol was developed to evaluate efficacy and find out whether patients can benefit from the procedure. In our study, we observed that patients with an LUS score of ASV ≥5.5 have a great likelihood of presenting a P/F ratio >300 mmHg on day 7, whereas patients with an LUS score of ASV ≥7 have quite a low risk of death. Therefore, the PLUE protocol can be used to predict the PPP in patients with ARDS.

PP response was assessed recently in two studies. Haddam et al. found that oxygenation response after PP was not correlated with a specific LUS pattern [5], whereas Prat et al. found that a normal LUS pattern of both anterobasal lung regions in supine position may predict a significant P/F ratio improvement [19]. One of the possible reasons for that difference was both of the studies chose oxygenation improvement as the index of the response, and we found that the oxygenation improvement did not correlate with the improvement in lung aeration. Ventilation was just one of the influential factors on oxygenation during PP; lung perfusion was also influenced after the change of body position. However, the dead space of the lung was also significantly reduced after the therapy, and the extent of the reduction was correlated with the LUS score. Gattinoni et al. found that patients with ALI/ARDS who responded to PP with reduction of their PaCO_2_ had increased survival at 28 days, in which the Ve(total minute ventilation)/PaCO_2_ value was used as a surrogate for the V_d_/V_t_ ratio [20]. In our study, the ASV in the LUS examination showed tight correlation with reduction of the dead space fraction, and the patients with ARDS who survived at 28 days had a significant increased ASV, so the ASV may be a good index to predict patient outcome.

Our study has a few limitations. The anterior chest wall was not included in our LUS protocol, so the aeration changes in these areas were not included in the ASV during PP. Moreover, LUS exclusively detected pulmonary foci extending to the visceral pleura. The foci decreased in size and did not extend to the visceral pleura during the therapy; thus, the foci could not be detected with LUS.

## Conclusions

LUS is a useful tool for monitoring the aeration changes of the lung-dependent area during PP. The PPP can be predicted in the patients with ARDS with the application of LUS.

## Abbreviations

ALI: Acute lung injury
APACHE: Acute Physiology and Chronic Health Evaluation
ARDS: Acute respiratory distress syndrome
ASV: Aeration score variation
AUC: Area under the ROC curve
AUROC: Area under the receiver operating characteristic curve
CT: Computed tomography
ETCO_2_: End-tidal carbon dioxide
FiO_2_: Fraction of inspired oxygen
ICU: Intensive care unit
LUS: Lung ultrasound
P/F: Ratio of partial pressure of arterial oxygen to fraction of inspired oxygen
PaCO_2_: Partial pressure of arterial carbon dioxide
PEEP: Positive end-expiratory pressure
PLUE: Prone position lung ultrasound examination
PP: Prone positioning
PPP: Prone positioning potential
TEE: Transesophageal echocardiography
V_d_: Dead space volume
V_t_: Tidal volume
